# Blood-Based Biomarkers in Traumatic Brain Injury: A Narrative Review With Implications for the Legal System

**DOI:** 10.7759/cureus.40417

**Published:** 2023-06-14

**Authors:** William R McBride, Nicholas R Eltman, Randel L Swanson

**Affiliations:** 1 Forensic Psychiatry, Rutgers Robert Wood Johnson Medical School, Piscataway, USA; 2 Center for Neurotrauma, Neurodegeneration and Restoration, Corporal Michael J. Crescenz Veterans Affairs Medical Center, Philadelphia, USA; 3 Physical Medicine and Rehabilitation, Rowan-Virtua School of Osteopathic Medicine, Stratford, USA; 4 Physical Medicine and Rehabilitation, University of Pennsylvania Perelman School of Medicine, Philadelphia, USA

**Keywords:** medical-legal, brain injury medicine, physical medicine and rehabilitation (pm&r), psychiatry, forensic medicine, biomarker, blood-based biomarker, brain injury, concussion, traumatic brain injury

## Abstract

Traumatic brain injury (TBI) is an increasingly recognized diagnosis with significant, and often costly, associated consequences. Yet, despite their increased recognition, TBIs remain underdiagnosed. This issue is especially prominent in the context of mild TBI (mTBI), where there often exists little to no objective evidence of brain injury. In recent years, considerable effort has been made to better define and interpret known objective markers of TBI, as well as identify and explore new ones. An area of particular interest has focused on research related to blood-based biomarkers of TBI. Advancements in our understanding of TBI-related biomarkers can make it possible to characterize the severity of TBI with greater accuracy, improve our understanding of staging within both the injury process and the recovery process, and help us develop quantifiable metrics representative of reversal and recovery from a brain injury following trauma. Proteomic and non-proteomic blood-based biomarkers are being studied extensively and have shown promise for these purposes. Developments in this realm have significant implications not only for clinical care but also for legislation, as well as civil and criminal litigation. Despite their substantial potential, most of these biomarkers are not yet ready for use within the clinical setting, and therefore, are not appropriate for use within the legal or policy-making systems at this time. Given that existing standardization for the accurate and reliable use of TBI biomarkers is currently insufficient for use within either the clinical or legal realms, such data can be vulnerable to misuse and can even result in the abuse of the legal system for unwarranted gain. Courts will need to carefully evaluate the information presented in their role as gatekeepers of the admissibility of scientific evidence within the legal process. Ultimately, the development of biomarkers should lead to improved clinical care following TBI exposure, coherent and informed laws surrounding TBI, and more accurate and just results in litigation surrounding TBI-related sequelae.

## Introduction and background

Traumatic brain injuries (TBIs) are responsible for an estimated 1.7 million emergency department visits, hospitalizations, and deaths per year in the United States [1]. This number does not account for the many people who suffer a TBI and do not seek treatment at all, or present to outpatient care in the days to weeks following the event. Contributing to an affected individual’s delay in, or lack of treatment-seeking behavior, is the high likelihood that if one experiences a TBI, it will be mild, resulting in milder, relatively short-lived symptoms. In fact, the Centers for Disease Control (CDC) estimates that 75% of TBIs are mild [2]. Despite the vast majority of TBIs being mild, the direct costs associated with TBI are estimated to be approximately 400 billion dollars each year worldwide [3]. In addition, it is estimated that among individuals who sustain a TBI, approximately 50,000 die each year from their injuries, and 80,000 to 90,000 suffer long-term disability [4].

History of TBI diagnosis and treatment in the United States

Military service puts individuals at higher risk of sustaining a TBI, even during peacetime [5]. TBIs have been common among those serving in the military throughout much of recent history, thus many of the advancements in TBI-related assessment and management have been motivated by the greater prevalence of need among those actively serving, or who have served in the armed forces.

Severe TBI was generally considered to be fatal before the 20th century [6,7]. During the Civil War, survival rates were known to be poor, mostly due to a high prevalence of infection. Penetrating head trauma was occurring at higher rates than in previous conflicts, largely secondary to the use of guns and explosives. Subdural and epidural hematomas, as well as diffuse axonal injuries, were described during this time period [6-8].

During World War I, advancements in antiseptic and neurosurgical techniques led to an improved mortality rate of 35% among those with head wounds and dural penetration [6-8]. This increase in survival rate prompted the need for the development of rehabilitation hospitals. Such development began in Germany and only advanced to the United States once the United States joined the World War I conflict [6,7].

By the advent of World War II, most inpatient TBI rehabilitation hospitals had closed; therefore, those who survived a TBI, yet continued to require TBI-related care, were commonly transferred to psychiatric hospitals [6,9]. Post-acute head injury rehabilitation programs with an interdisciplinary treatment focus were first established around this time period [6,10]. Most of these programs were again shut down after the conclusion of World War II until the need for them was revived by the commencement of the Korean War. Helmets were made mandatory in war zones for United States soldiers during this era [6,11]. Furthermore, mobile teams were established to rapidly evacuate hematomas, resulting in a significant decrease in mortality [6,12].

Around the time of the Vietnam War, increased civilian access to high-speed transportation options led to an increase in TBI diagnoses within the United States [6,13]. Concomitantly, approximately 14% of the survivors of the war had suffered a TBI [6,14,15]. These developments led to an increase in overall demand for (and subsequently an increase in) the availability of care for TBI patients in both private and military hospitals [6].

In the Gulf War, and, subsequently, Operation Iraqi Freedom and Operation Enduring Freedom, TBI became so prevalent that it was described as the signature wound of these conflicts [6]. This is likely due in part to advancements in blast-related technologies and an increase in the use of explosive devices and high-powered ammunition. It is estimated that approximately 20% of veterans who deployed over this time period experienced at least one mild TBI exposure and that greater than 50,000 veterans are currently living with a history of blast-related TBI [5,6,16,17]. Approximately 20% of those who suffered any injuries during these campaigns are believed to have sustained a TBI [18].

Current issues in TBI medicine

In recent decades, advancement in TBI-related neuropathology, neuroimaging, and blood-biomarker research has captured the attention of the public, the press, and politicians. However, these advancements have not yet translated into clinical applications, and while TBI remains a significant issue in the United States and abroad, existing clinical TBI classification systems have shown limited prognostic value, diagnostic value, or research utility, especially in the setting of mild TBI (mTBI) [19]. With increased public attention being given to mTBI, the number of lawsuits involving alleged TBI exposure has roughly tripled over the past 20 years [20,21]. Such cases can be particularly challenging because there is often no objective evidence of brain injury in cases of mTBI exposure. Importantly, the diagnostic classification of mTBI severity is often based on self-reported details of the traumatic event, along with acute and sub-acute symptoms (Table 1) [22,23]. Such symptoms often include confusion, photophobia, tinnitus, dizziness, headaches, low mood, fatigue, short-term memory loss, difficulty concentrating, and irritability [23].

**Table 1:** Classification of TBI severity. *: If a patient meets the criteria in more than one category of severity, a higher severity level is assigned. **: No clinically relevant findings. ***: Alteration of mental status must be immediately related to the trauma to the head. Typical symptoms would be looking and feeling dazed and uncertain of what is happening, confusion, difficulty thinking clearly or responding appropriately to mental status questions, and/or being unable to describe events immediately before or after the trauma injury event. ****: In April 2015, the Department of Defense released a memorandum recommending against the use of Glasgow Coma Scale scores to diagnose TBI. Note: The table was reproduced from the *VA/DoD Clinical Practice Guideline for the Management and Rehabilitation of Post-Acute Mild Traumatic Brain Injury (mTBI)*,which is a document jointly developed and approved by the U.S. Department of Veterans Affairs (VA) and U.S. Department of Defense (DoD), and which is freely available in the public domain, as detailed in the following reference [23]. TBI: traumatic brain injury

Criteria*	Mild*	Moderate*	Severe*
Structural imaging	Normal**	Normal or abnormal	Normal or abnormal
Loss of consciousness	0–30 minutes	>30 minutes and <24 hours	>24 hours
Alteration of consciousness/Mental state***	Up to 24 hours	>24 hours; severity based on other criteria	>24 hours; severity based on other criteria
Post-traumatic amnesia	0–1 day	>1 and <7 days	>7 days
Glasgow Coma Scale (best available score in first 24 hours)****	13–15	9–12	<9

Many of these symptoms overlap with those of posttraumatic stress disorder (PTSD), depression, anxiety, migraine headaches, and other conditions [22,23]. In approximately 10% of those diagnosed with mTBI, symptoms persist beyond three months status post-injury [24]. This phenomenon is known as post-concussive syndrome (PCS). Risk factors for the development of PCS include pre-existing depression, PTSD, anxiety, and certain personality types (high achieving, dependent, or insecure) [24]. In addition, higher acute symptom scores following the event, early exhibition of emotional symptoms, and migraine headaches have been identified as risk factors for the development of PCS [25]. Because there is often a lack of objective evidence for mTBI within the post-concussion syndrome cohort, multiple questions can be raised: Are the symptoms more attributable to a pre-existing or separate illness? Are the symptoms being reported for secondary gain? Are the objective measures being utilized (such as blood-based biomarkers, neurological imaging techniques, and neuropsychological testing) sensitive enough to detect abnormalities related to mTBI?

Advancements in our understanding of TBI-related biomarkers can make it possible to more accurately characterize the severity of TBI, improve our understanding of staging within both the injury process and the recovery process, and help us develop quantifiable metrics representative of reversal and recovery from a brain injury following trauma [19]. These developments will have dramatic implications not only for clinical care but also for legislation, as well as civil and criminal litigation. The following narrative review will discuss the developing research surrounding blood-based biomarkers with relevance to mTBI and, subsequently, the potential impact that the development of such biomarkers could have within the legal system.

## Review

Biomarkers of mTBI

Over the past 20 years, there has been a substantial increase in the amount of TBI-related blood-based biomarker studies conducted; however, there remains a dearth of reliable blood biomarkers available for clinical use. This is due to a combination of factors including, but not limited to, the inherent heterogeneity of TBI exposure (e.g., differing injury severity, mechanism of injury, anatomical location injured), differing pathophysiological endophenotypes, and neurological and psychiatric comorbidities that may pre-date or be co-morbid with TBI exposure [19]. For classification, these biomarkers are divided into proteomic and non-proteomic biomarkers.

Proteomic Biomarkers

Proteomic (protein-based) biomarkers have been the main focus of TBI blood-based biomarker research over the past 20 years. One of the most extensively studied TBI-related biomarkers to date is S100 calcium-binding protein B (S100B) [19]. It has been proposed as a potential predictor for the necessity of performing neuroimaging shortly after TBI exposure, as elevated S100B levels have been associated with amplified glial activation, progression of the secondary injury process, and a deteriorated prognosis following TBI [19,26,27]. There exist multiple limitations with regard to the application of this biomarker, however. These limitations include a lack of neuronal specificity (S100B levels are also increased in response to physical exertion and polytrauma), a short time frame following TBI exposure of three to six hours within which the level must be collected and measured, and a lack of congruity among what are considered normal values between different age groups [19,27-32]. Overall, there is minimal evidence to suggest that blood levels of S100B outperform emergency evaluations of TBI by trained practitioners [19,32,33]. Thus, other TBI-related proteomic blood biomarkers have been investigated, including neurofilament light (NfL), ubiquitin C-terminal hydrolase (UCH-L1), glial fibrillary acidic protein (GFAP), and tau [19].

Considerable research has demonstrated that NfL levels can be helpful in determining the severity of TBI, both in the acute phase and the months to years following injury [19,27,34-38]. Increasing serum NfL has demonstrated associations with the progression of diffuse axonal injury (DAI) longitudinally post-TBI exposure, delayed recovery, and worsened patient outcomes (including post-concussive symptoms persisting for more than a year post-mTBI exposure [19,27,34,35,39,40]. There is even limited evidence suggesting that, in healthy controls, elevated NfL levels are unchanged following exercise [19,34]. Thus, there appears to be a growing body of evidence suggesting that serum NfL may be a more versatile and more useful biomarker than S100B.

UCH-L1 appears to be useful in cases of severe TBI [19,35,37,41]. This protein is upregulated following neuronal damage [19,27,42]. Elevated levels of UCH-L1 have been found in patients who have findings consistent with DAI on neuroimaging when compared with patients who suffered focal injuries [19,37,41]. Elevated levels also appear to be associated with increased mortality and worse long-term memory performance [19,37,43,44]. Importantly, this biomarker has limited to no value when used alone in cases of mild-to-moderate TBI [19,35].

GFAP is a protein that is upregulated during astrogliosis in response to central nervous system (CNS) injury [19,27]. Some studies have suggested that it can help predict the need for neuroimaging and neurosurgical intervention following TBI [19,37,39,45]. Notably, the clinical utility of using both GFAP and UCH-L1 together appears to be promising for the purposes of documenting mTBI and predicting the necessity of neuroimaging after mTBI exposure [19,27,39,46].

Other potentially useful proteomic biomarkers include tau, alpha-II spectrin, amyloid beta, neuron-specific enolase, neurofilament heavy chain, and high-sensitivity C-reactive protein (hs-CRP), among others; however, the evidence for clinical utility is currently inconclusive for each of these potential biomarkers when considered individually [19]. However, there is preliminary evidence to suggest that protein panels may be more useful as TBI biomarkers than any one serum protein alone [19,37].

Disadvantages of proteomic biomarkers include a particular susceptibility to rapid decomposition in the bloodstream (short half-life), variable degrees of enrichment within the brain, variability in expression across different regions of the brain, and extraneuronal expression (proteins may be expressed with exercise, polytraumatic injuries, or other confounding variables) [19,27,47]. Given the general limitations of the use of proteins as blood biomarkers, researchers are beginning to examine non-protein biomarkers following TBI exposure [27,47].

Non-proteomic Biomarkers

Recent research has suggested reasons for optimism pertaining to the potential diagnostic and prognostic utility of non-proteomic biomarkers following TBI exposure [19]. Extracellular vesicles and micro-RNAs are two of the most promising non-proteomic biomarkers [19,27].

Extracellular vesicles (EVs) are lipid-bound organelles secreted from cells, whose contents can vary based on their specific cellular origin and the condition of the cell [19,27,48]. Given their membrane encapsulation, EVs have an inherent capacity to protect internal protein components from degradation and are considered to be easily identifiable in the peripheral bloodstream [19,49]. Detection and quantification of the cargo proteins within EVs are being investigated for their potential utility in TBI recovery in both the acute and chronic phases [19,50-56]. Early research suggests potential viability for their use across the spectrum of TBI severity, with specific regard to both diagnosis and prognosis [19,27].

MicroRNAs are small, single-stranded, non-coding RNA molecules [57]. They are involved in RNA silencing and post-transcriptional regulation of gene expression [58,59]. MicroRNAs are necessary for a multitude of CNS processes, including differentiation and maturation of neurons, as well as the formation and pruning of synapses [19,27,60,61]. Both TBI exposure and other neurodegenerative diseases have been associated with irregular expression of microRNAs within the CNS [19,27,56,60-66]. Potential advantages of the use of microRNAs in this context include their high expression within the brain and their resistance to degradation in peripheral biofluids, both as a cargo component of EVs and as freely circulating molecules [19,32,61]. MicroRNAs appear to have the potential for use in severe TBI for both diagnosis and prognosis; however, their utility in mild and moderate TBI is inconclusive at this time [19,27,56,60,62-66].

The goals of developing and better understanding these biomarkers include improving current diagnostic and prognostic accuracy while accounting for variations in serum biomarker concentration over the time course of post-TBI exposure [35], confirming the presence or absence of mTBI, discerning which TBI exposures require acute neuroimaging and/or neurosurgical assessment, predicting delayed recovery after mTBI, revealing specific TBI-induced neuropathological endophenotypes, predicting long-term outcomes, and identifying patients who could benefit from targeted treatments [19].

Potential legal implications

Advancements in neuroscience have led to changes in legal policy in the past. In a series of cases from 2005 to 2012, the United States Supreme Court determined that an execution or an automatic sentence of life without parole for someone younger than 18 years old at the time of a crime violated the 8th Amendment prohibition against cruel and unusual punishment [20,67-69]. This ruling relied upon research findings that indicated that the human brain does not develop fully until the mid-20s. Inherently, almost every major scientific advancement brings with it new challenges for the legal system. Who qualifies as a true expert regarding novel subject matter? What evidence is admissible in court? Is there a different threshold for admissibility in criminal versus civil cases? These questions and many others must be answered, often without complete information during litigation and policy development.

Legal decisions are significantly more binary by nature than those of science, where it is commonly accepted that at any point in time, something may not be completely known about a topic yet. Verdicts must be rendered based on the evidence that is present. This information is of great impact to those involved in the case and, therefore, must be both applicable and well-established. Therefore, standards of evidence regarding what should be considered admissible within the legal system have been developed to best utilize expert testimony. There exist two standards for the admissibility of evidence in the United States: The *Frye Standard* and the *Daubert Standard* [70-72].

The *Frye Standard* was articulated in DC Circuit Court in 1923 in *Frye vs. United States* [73,74]. The court held that using systolic blood pressure as evidence that an individual is lying did not reach the threshold of acceptance within the scientific community and was therefore not admissible. This judgment was not based on reliability or reproducibility but instead on whether or not the method or scientific principle at issue was “sufficiently established to have gained general acceptance in the particular field in which it belongs” [73,74]. One of the persistent difficulties related to *Frye* is the notion of “general acceptance.” This issue can be especially difficult when the concept of “general acceptance” pertains to recent advancements or new technology. The *Frye Standard *was the predominant standard for decades across the United States; however, it is no longer the standard federally, or in 42 of the 50 states. California, Illinois, Maryland, Minnesota, New York, New Jersey, Pennsylvania, and Washington still use the *Frye Standard* statewide [20].

The *Frye Standard *was superseded by the landmark United States Supreme Court case of *Daubert v. Merrell Dow* (1993) [75] and two separate later cases that elaborated on the *Daubert Standard* [75-78]. This standard articulated the new federal framework for the admissibility of expert scientific testimony based on an updated interpretation of the Federal Rules of Evidence. Under the *Daubert Standard*, the factors that can be considered in determining whether a methodology is valid are: whether the theory or technique in question can be and has been tested; whether it has been subjected to peer review and publication; the theory or technique’s known or potential error rate; the existence and maintenance of standards controlling the theory or technique’s operation; and whether the theory or technique has attracted widespread acceptance within a relevant scientific community [20,76-79].

Establishing a standard of evidence with broad and explicitly flexible guidelines, such as the *Daubert Standard*, has advantages and disadvantages, particularly when attempting to apply and interpret scientific evidence [20]. This issue is amplified when discussing recent advancements such as blood-based biomarkers or neuroimaging and mTBI. While there may be insufficient data available to meet optimal standardization parameters at this time, there still exists a high demand for their use in the legal decision-making process.

The promise of further articulating objective signs of brain injury makes TBI-related biomarkers a potentially attractive and useful tool within the legal realm, with the potential to address many of the ambiguities surrounding mTBI litigation. One of the primary areas of impact that the acceptance of TBI-related biomarkers will bring about is that of tortious claims. As described by the *Legal Information Institute* of Cornell Law School, “a tort is an act or omission that gives rise to injury or harm to another and amounts to a civil wrong for which courts impose liability,” and the “primary aims of tort law are to provide relief to injured parties for harms caused by others, to impose liability on parties responsible for the harm, and to deter others from committing harmful acts” [80]. The necessary components for recovery of damages in tort are duty, breach of duty, causation, and damages. In *Biomarkers, Concussions, and the Duty of Care*, Grey et al. opined that the development and acceptance of TBI-related biomarkers could substantially inform causal issues in tort lawsuits, as well as duties, and could even assist in substantiating latent injury claims through an enhanced ability to investigate long-term effects and injuries [70].

A pertinent and highly publicized example of a realm that would be greatly affected by these developments is professional and non-professional sports. Legal responsibilities in the diagnosis and management of youth sports-related mTBIs would probably shift to those that are more directly involved in the player’s participation, such as a combination of trainers, schools, physicians, parents, and the players themselves [70]. This will likely necessitate an updated public policy discussion pertaining to these issues [70].

Typical duty within the sports domain currently consists of the responsibility of leagues and/or venues to inform the athletes of the risk of participation, provide equipment for, and implement appropriate rules for the event or activity at hand [70]. Furthermore, duty often exists for the leagues and/or venues to accurately diagnose mTBI, remove participants as appropriate, and not allow their return to play until the affected participants are determined to be ready [70]. These standards originate from a mix of common law and legislative evolution [70].

Individual states have taken the lead role in the United States regarding return-to-play determinations in youth sports; however. there is no federal law regulating these determinations [70]. The majority of states have laws surrounding removal from and return to play. Determination of who is responsible for making these decisions varies from state to state (e.g., trainer vs. coach vs. physician) [70]. Identification of appropriate blood-based biomarkers could lead to more uniform legislation among states, with the probable development of a minimum standard for prevention and management [70]. Grey et al. projected that such legislation could potentially incorporate mandatory baseline testing being conducted before a season; schools being required to report mTBIs to a centralized registry; mandatory monitoring of biomarker levels throughout a season; and potentially even acute post-concussion testing and subsequent monitoring for determining return to play [70]. They opine that “such legislation would likely include civil liability and/or penalties for non-compliance” [70].

This may encourage the proliferation of private claims made in professional malpractice lawsuits and negligence claims against the organization responsible for the athletic event [70]. Grey et al. described the duty and breach issues that could arise in these lawsuits to include a failure to establish a baseline (functional status and/or biomarker level), failure to conduct appropriate screening, misdiagnosis of mTBI, and premature determination that an individual is ready to return to play (compared to objective biomarker levels indicating that the individual is not ready to return) [70]. The use of biomarkers could significantly change the return-to-play process and decrease the uncertainty associated with medical clearance and mTBI [70]. In addition, a participant’s subjective report of their experience of an injury would likely become more objectively verifiable with the development of pertinent biomarkers, and their own consent to return to play significantly better informed [70]. Of course, the direction of further advancements within the field of study of TBI will create new questions for investigation: If there was a biomarker that predicted susceptibility to TBI or damage from a TBI exposure, should the participant’s ability to consent to participate need to meet a higher standard? Should one be allowed to participate if blood-based biomarkers indicate that they are especially vulnerable to long-term CNS damage [70]? Pertinent blood-based biomarkers could even have a significant impact on claims for chronic traumatic encephalopathy, especially if an individual is able to provide evidence that an injury did, or did not, occur [70]. The above examples encapsulate a fraction of what will likely be a broad swath of exciting, challenging, and illuminating legal and ethical questions that will be raised alongside the advancement of TBI-related biomarkers.

## Conclusions

As a society, we exist within a potentially paradigm-shifting moment with regard to our understanding of, and response to, TBI exposures. The current lack of sufficiently objective tests for diagnosis, prognosis, and monitoring of TBIs, as well as our inability to accurately and reliably measure individual susceptibility and response to TBIs, are impeding the development of effective policies aimed at preventing, assessing, and treating TBIs, as well as allocating responsibility when they occur. While objective signs of TBI that could impact diagnosis, prognosis, and treatment are being defined, the vast majority are not yet ready for use within the clinical setting. Furthermore, despite their promise, most of them are not appropriate for use within legal or policy-making systems at this time. Given that current standardization for the accurate and reliable use of TBI blood biomarkers is insufficient in both the clinical and legal realms, such data could be vulnerable to misuse, and could even result in the abuse of the legal system for unwarranted gain. Courts will be tasked with the challenge of determining the admissibility of related scientific evidence throughout this process. Ultimately, the development of biomarkers should lead to improved clinical care for those who have suffered a TBI, coherent and informed laws surrounding TBI, and more accurate and just results in litigation surrounding TBI-related injuries.
